# LncRNA LOC146880 promotes esophageal squamous cell carcinoma progression via miR-328-5p/FSCN1/MAPK axis

**DOI:** 10.18632/aging.203037

**Published:** 2021-05-18

**Authors:** Jianwei Tang, Honglei Xu, Qiang Liu, Jianan Zheng, Cheng Pan, Zhihua Li, Wei Wen, Jun Wang, Quan Zhu, Zhibo Wang, Liang Chen

**Affiliations:** 1 Department of Thoracic Surgery, The First Affiliated Hospital of Nanjing Medical University, Nanjing 210029, Jiangsu Province, China

**Keywords:** esophageal squamous cell carcinoma, LOC146880, miR-328-5p and FSCN1, H3K27 acetylation, MAPK signaling pathway

## Abstract

We investigated the role of long non-coding RNA (lncRNA) LOC146880 in esophageal squamous cell carcinoma (ESCC). LOC146880 was significantly upregulated in ESCC tissues (*n* = 21) and cell lines compared to the corresponding controls. Higher LOC146880 expression correlated with poorer overall survival (OS) of ESCC patients. Moreover, CREB-binding protein (CBP) and H3K27 acetylation levels were significantly higher in the LOC146880 promoter in ESCC cell lines than in the controls. LOC146880 silencing inhibited *in vitro* proliferation, invasion, migration, and epithelial-mesenchymal transition of ESCC cells. LOC146880 silencing also induced G1-phase cell cycle arrest and apoptosis in ESCC cells. Bioinformatics analysis, dual luciferase reporter assays, and RNA immunoprecipitation assays showed that LOC146880 regulates FSCN1 expression in ESCC cells by sponging miR-328-5p. Moreover, FSCN1 expression correlated with activation of the MAPK signaling pathway in ESCC cells and tissues. *In vivo* xenograft tumor volume and liver metastasis were significantly reduced in nude mice injected with LOC146880-silenced ESCC cells as compared to those injected with control shRNA-transfected ESCC cells. These findings show that the LOC146880/miR-328-5p/FSCN1/MAPK axis regulates ESCC progression *in vitro* and *in vivo*. LOC146880 is thus a promising prognostic biomarker and potential therapeutic target in ESCC.

## INTRODUCTION

Esophageal cancer (EC) is the seventh most common cancer type and sixth leading cause of cancer-related deaths worldwide [1]. The two major histological subtypes of EC are esophageal squamous cell carcinoma (ESCC) and esophageal adenocarcinoma (EAC). ESCC accounts for more than 90% of EC cases in China [2]. With the prevalence of neoadjuvant chemoradiotherapy, the five-year survival rate of ESCC is greater than 30% [3]. There is thus an urgent need to identify novel prognostic biomarkers and therapeutic targets associated with ESCC progression.

Long non-coding RNAs (LncRNAs) are a group of non-coding RNAs that are longer than 200 nucleotides. They play a significant role in tumorigenesis by regulating the expression or function of their target genes through epigenetic transcriptional regulation, protein modifications, or formation of lncRNA-protein complexes [4–9]. LncRNAs regulate expression levels of specific miRNA-targeted genes by acting as sponges for their target microRNAs (miRNAs) [10, 11]. Previous studies have demonstrated the critical role of lncRNAs in ESCC progression. For example, lncRNA sponging of miR-224, TUSC7 suppresses chemotherapeutic resistance in ESCC cells by modulating the DESC1/EGFR/AKT pathway [12]. And by sponging miR-214, LncRNA HNF1A-AS1 promotes ESCC by upregulating SOX-4 expression [13].

LOC146880 is a *Rho GTPase activating protein 27 pseudogene 1* or *ARHGAP27P1*-derived lncRNA, which is located at chromosome 17q24.1; genetic variants in this region are associated with increased susceptibility of prostate and lung cancers [14, 15]. Pseudogene-derived lncRNAs modulate biological functions similar to other classical lncRNAs, and play a vital role in cancers such as ESCC [16]. Pseudogene-derived lncRNAs such as PHBP1, PTTG3P, and FTH1P3 function as tumor promoters or tumor suppressors in ESCC [17–19].

A previous study showed that high expression of LOC146880 was significantly associated with worse prognosis of lung cancer patients [20]. Deng et al showed that PM_2.5_ exposure enhanced epithelial-mesenchymal transition (EMT) of lung cancer cells by increasing the levels of reactive oxygen species (ROS), autophagy, and LOC146880 [21]. These findings suggested that LOC146880 functioned as a tumor promoter in lung cancer. Conversely, LOC146880 was significantly downregulated in gastric cancer tissues; LOC146880 overexpression significantly reduced their proliferation, invasion, and migration of gastric cancer cells [22]. This suggested that LOC146880 played contradictory roles in lung and gastric cancers. The role of LOC146880 in ESCC is not well studied. Therefore, in this study, we investigated the role of LOC146880 in ESCC growth and progression.

## RESULTS

### LOC146880 is overexpressed in ESCC tissues and cells

**Figure 1:**
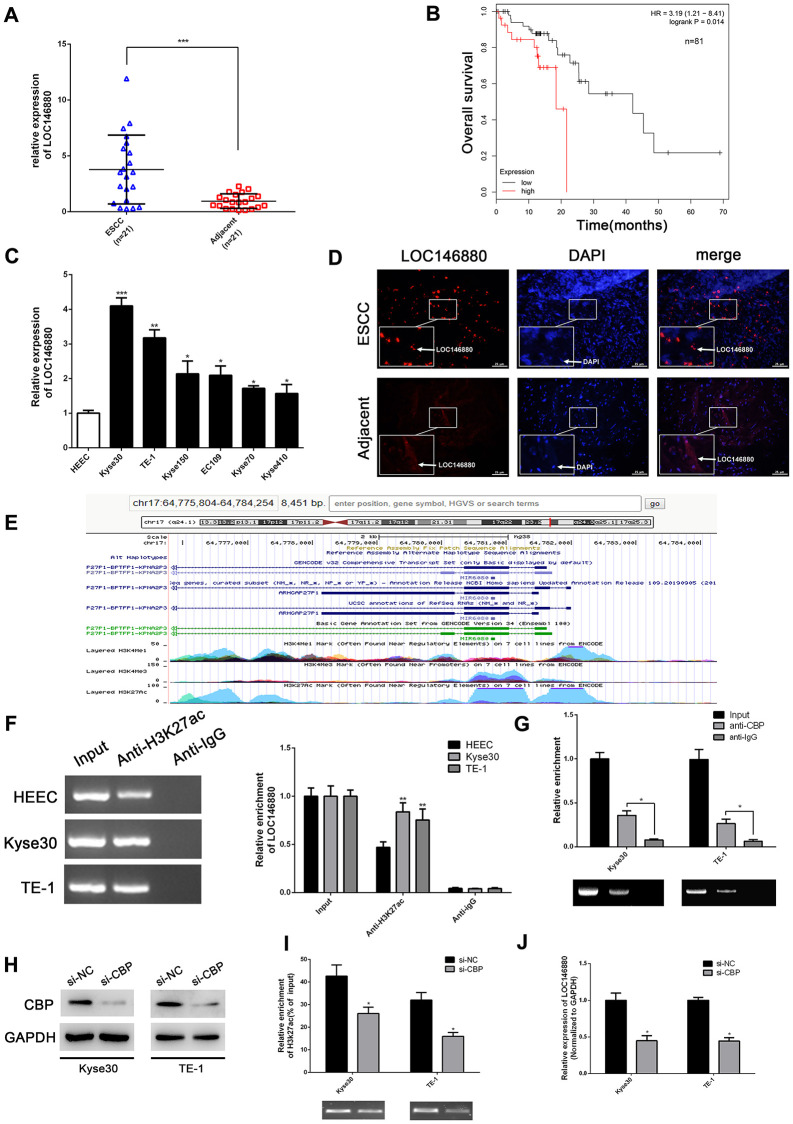
**LOC146880 expression is upregulated in ESCC tissues and cells.** (**A**) The expression levels of LOC146880 in 21 pairs of ESCC and adjacent normal esophageal tissues. (**B**) Kaplan-Meier survival curve analysis shows overall survival of ESCC patients with high- and low-expression levels of LOC146880 (*n* = 81). (**C**) QRT-PCR analysis shows LOC146880 expression levels in human esophageal epithelial cells and ESCC cell lines (EC109, TE-1, Kyse30, Kyse70, Kyse150, and Kyse410). (**D**) FISH analysis shows LOC146880 expression in ESCC and normal esophageal tissues. (**E**) Bioinformatics analysis (https://genome.ucsc.edu/) shows H3K27ac levels in the promoter region of LOC146880. (**F**) ChIP assay analysis with anti- H3K27ac antibody shows H3K27ac levels in the LOC146880 promoter of HEEC, Kyse30, and TE-1 cells. (**G**) ChIP assay analysis with anti-CBP antibody shows CBP protein levels in the LOC146880 promoter of HEEC, Kyse30, and TE-1 cells. (**H**) Western blot analysis shows CBP protein levels in control and CBP-silenced Kyse30, and TE-1 cells. (**I**) ChIP assay analysis shows H3K27ac levels in the LOC146880 promoter of control and CBP-silenced Kyse30, and TE-1 cells. (**J**) QRT-PCR analysis shows LOC146880 expression levels in control and CBP-silenced Kyse30 and TE-1 cells. ^*^P < 0.05, ^**^P < 0.01, ^***^P < 0.001.

The expression of LOC146880 was significantly upregulated in ESCC tissues compared to adjacent normal esophageal tissues (*n* = 21; Figure 1A). Kaplan-Meier survival analysis showed that overall survival of patients with high LOC146880 expression was significantly lower than those with low LOC146880 expression (Figure 1B). Moreover, expression of LOC146880 was significantly higher in ESCC cell lines (EC109, TE-1, Kyse30, Kyse70, Kyse150 and Kyse410) compared to the human esophageal epithelial cells (HEECs) (Figure 1C). The expression of LOC146880 was highest in Kyse30 and TE-1 cells among the ESCC cell lines (Figure 1C). Hence, Kyse30 and TE-1 cells were chosen for further experiments. FISH analysis confirmed LOC146880 was overexpression in Kyse30 and TE-1 cells (Figure 1D).

### LOC146880 is transcriptionally activated in ESCC by H3K27 acetylation and CBP

Furthermore, we explored the mechanisms that promote higher LOC146680 expression levels in ESCC cells. Bioinformatics analysis (https://genome.ucsc.edu/) showed enrichment of H3K27ac levels in the promoter region of LOC146880 (Figure 1E). ChIP assay results confirmed that H3K27ac levels were higher in the LOC146880 promoter region of Kyse30 and TE-1 cells compared to the HEEC cells (Figure 1F). Previous studies have shown that CREB-binding protein (CBP) plays a key role in chromatin acetylation [23]. Therefore, we estimated CBP protein levels in the LOC146680 promoter region of ESCC and HEEC cells. ChIP assay with anti-CBP antibody showed that CBP protein levels were significantly higher in the LOC146680 promoter region of both Kyse30 and TE-1 cells compared to the HEEC cells (Figure 1G). Furthermore, H3K27ac levels were significantly reduced in the LOC146680 promoter region of CBP-silenced ESCC cells (Figure 1H, 1I). This correlated with reduced LOC146880 expression in CBP-silenced ESCC cells (Figure 1J). Taken together, these results showed that increased CBP-mediated histone acetylation in the LOC146880 promoter upregulated the levels of LOC146880 in ESCC tissues and cells.

### LOC146880 silencing decreases proliferation, survival, and EMT of ESCC cells

**Figure 2:**
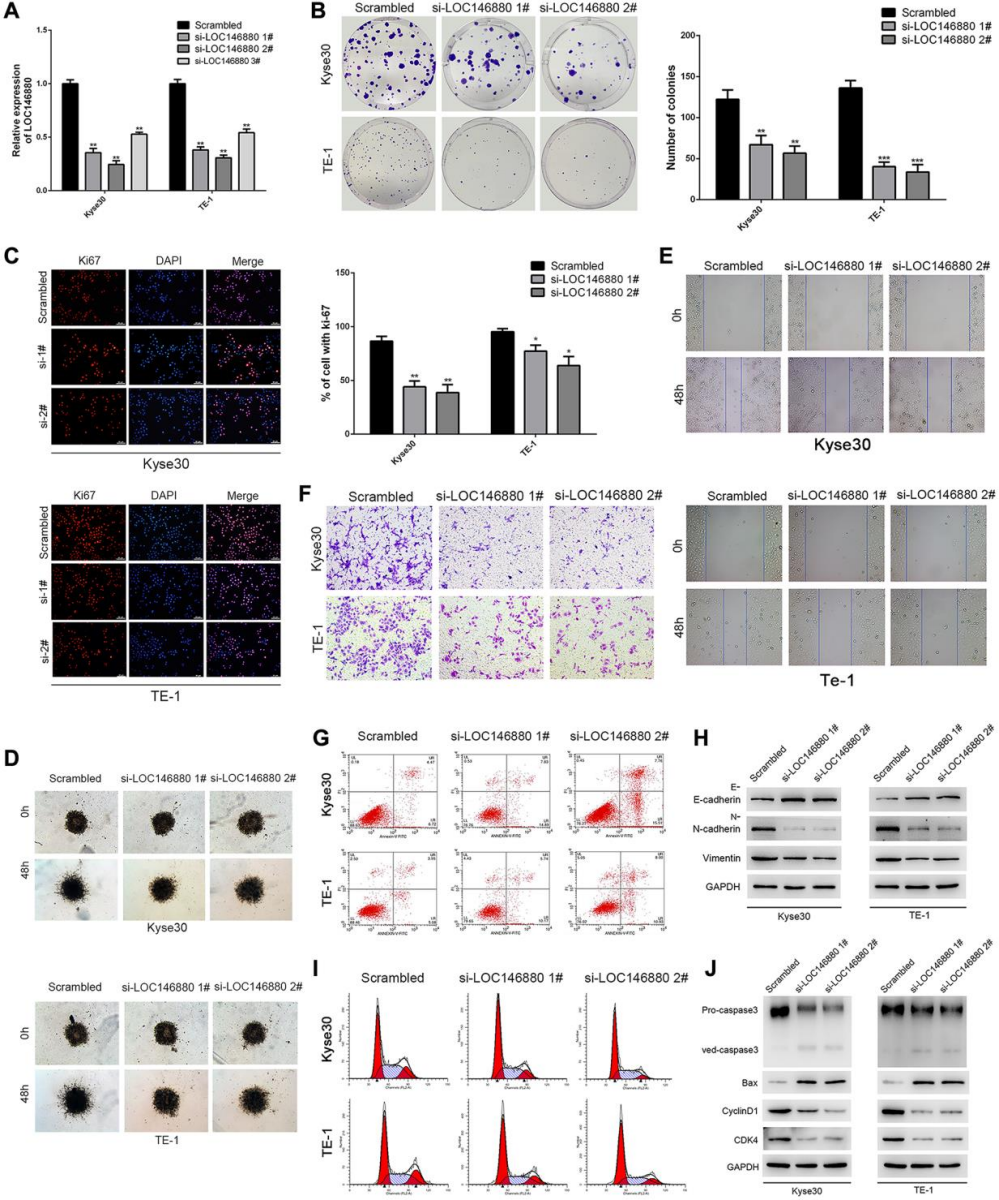
**Knockdown of LOC146880 inhibits growth and progression of ESCC cells.** (**A**) QRT-PCR analysis shows LOC146880 expression levels in ESCC cells transfected with si-NC (scrambled control siRNA), si-LOC146880#1, si-LOC146880#2, and si-LOC146880#3. (**B**) Colony formation assay results show viability of Kyse30 and TE-1 cells respectively transfected with si-NC, si-LOC146880#1, or si-LOC146880#2. (**C**) Immunofluorescence assay results show Ki-67 expression levels in control and LOC146880-silenced Kyse30 and TE-1 cells. (**D**) 3-dimensional spheroid assay results show the migration ability of control and LOC146880-silenced Kyse30 and TE-1 cells. (**E**) Wound healing assay results show the migration ability of control and LOC146880-silenced Kyse30 and TE-1 cells. (**F**) Transwell assay results show the invasiveness of control and LOC146880-silenced Kyse30 and TE-1 cells. (**G**) Flow cytometry analysis shows apoptotic rates of control and LOC146880-silenced Kyse30 and TE-1 cells. (**H**) Western blot analysis shows expression levels of E-cadherin (epithelial cell marker) as well as N-cadherin and vimentin (mesenchymal cell markers) in control and LOC146880-silenced Kyse30 and TE-1 cells. (**I**) Flow cytometry analysis shows cell cycle distribution of control and LOC146880-silenced Kyse30 and TE-1 cells. (**J**) Western blot analysis shows the levels of pro-apoptotic proteins (cleaved caspase-3 and Bax) and cell cycle proteins (cyclinD1 and CDK4) in control and LOC146880-silenced Kyse30 and TE-1 cells. ^*^P < 0.05, ^**^P < 0.01, ^***^P < 0.001.

We transfected Kyse30 and TE-1 cells with si-NC (scrambled control), si-LOC146880 #1, si-LOC146880 #2, and si-LOC146880 #3 to knockdown the expression of LOC146880. QRT-PCR analysis showed that the expression levels of LOC146880 were significantly reduced in si-LOC146880#1 and si-LOC146880#2-transfected Kyse30 and TE-1 cells compared to si-NC-transfected Kyse30 and TE-1 cells (Figure 2A). Therefore, we used si-LOC146880 #1 and si-LOC146880 #2 for further experiments. LOC146880 silencing significantly reduced the colony formation ability of Kyse30 and TE-1 cells (Figure 2B). LOC146880 knockdown significantly reduced Ki67 protein levels in Kyse30 and TE-1 cells (Figure 2C). Moreover, knockdown of LOC146880 significantly reduced migration and invasiveness of Kyse30 and TE-1 cells (Supplementary Figure 1A, 1B; Figure 2D–2F). Furthermore, LOC146880 knockdown significantly increased E-cadherin (epithelial cell marker) and significantly reduced N-cadherin and vimentin (mesenchymal cell markers), in Kyse30 and TE-1 cells (Figure 2H). Flow cytometry analysis showed increased apoptosis of LOC146880 knockdown ESCC cells compared to si-NC-transfected ESCC cells (Figure 2G and Supplementary Figure 1C). LOC146880 silencing induced G1 phase cell cycle arrest in Kyse30 and TE-1 cells (Figure 2I and Supplementary Figure 1D). LOC146880 silenced ESCC cells showed significantly higher expression of pro-apoptotic proteins (cleaved Caspase-3 and Bax) and significantly lower expression of G1-phase-promoting proteins (cyclinD1 and CDK4) (Figure 2J). These results demonstrate that LOC146880 regulates proliferation, survival, and EMT in ESCC cells.

### LOC146880 sponges miR-328-5p in ESCC cells

**Figure 3:**
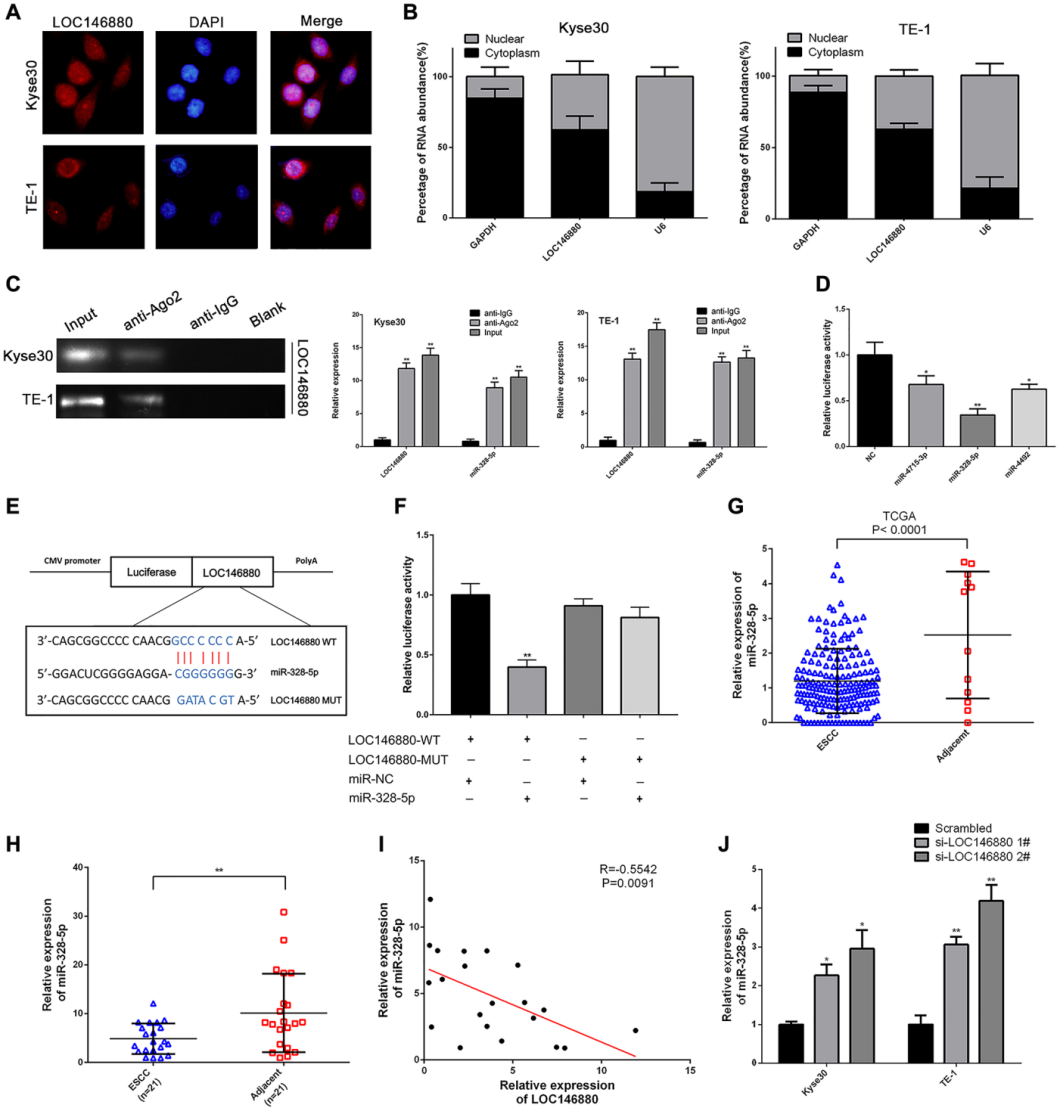
**LOC146880 sponges miR-328-5p in ESCC cells.** (**A**) FISH analysis shows localization of LOC146880 (green) in the cytoplasm and nucleus (blue) of Kyse30 and TE-1 cells. (**B**) Relative levels of LOC146880 in the nuclear and cytosolic fractions of Kyse30 and TE-1 cells. U6 and GAPDH were used as controls for the nuclear and cytosolic fractions. (**C**) RIP assay shows fold enrichment of LOC146880 and miR-328-5p in the anti-Ago2-antibody and IgG control groups. (**D**) Dual luciferase reporter assay results show relative luciferase activity of Kyse30 cells co-transfected with vector containing LOC146880-WT plus vectors containing one of the three miRNAs (miR-4715-3p, miR-328-5p and miR-4492). (**E**) Predicted miR-328-5p seed region in the wild-type (WT) and mutated (Mut) LOC146880. (**F**) Dual luciferase reporter assay shows relative luciferase activity in Kyse30 cells co-transfected with luciferase reporter plasmid containing wild type (WT) or mutant (Mut) LOC146880 and miR-328-5p mimics, miRNA-328-5p NC act as control. (**G**) TCGA database analysis shows expression levels of miR-328-5p in ESCC (*N* = 183) and adjacent normal esophageal tissues (*N* = 12). (**H**) QRT-PCR analysis shows expression levels of miR-328-5p in 21 pairs of ESCC and paracancerous esophageal tissues. (**I**) Spearman’s rank correlation analysis shows inverse relationship between LOC146880 and miR-328-5p expression levels in the 21 pairs of ESCC and paracancerous esophageal samples. (**J**) QRT-PCR analysis shows expression levels of miR-328-5p in control and LOC146880 silenced Kyse30 and TE-1 cells. ^*^P < 0.05, ^**^P < 0.01, ^***^P < 0.001.

Next, we analyzed the mechanism through which LOC146880 promoted ESCC progression. FISH assay analysis showed that LOC146880 was more abundant in the cytoplasm than in the nucleus of ESCC cells (Figure 3A, 3B). This suggested that LOC146880 regulated target gene expression at the post-transcriptional level. RIP assay with anti-Ago2-antibody showed enriched binding of LOC146880 and miR-328-5p (Figure 3C). This suggested that LOC146880 potentially interacted with miRNAs such as miR-328-5p. Starbase2 database (http://starbase.sysu.edu.cn/) analysis identified potential miR-4715-3p, miR-328-5p, and miR-4492 binding sites in the LOC146880 sequence. Dual luciferase reporter assay showed that all three miRNAs (miR-4715-3p, miR-328-5p and miR-4492) suppressed luciferase activity from the luciferase vector containing wild-type LOC146880, with miR-328-5p showing the strongest effect (Figure 3D). Furthermore, we constructed recombinant luciferase reporter plasmids with wild-type LOC146880 (LOC146880-WT) or mutated LOC146880 (LOC146880-MUT) (Figure 3E). Dual luciferase reporter assay showed that miR-328-5p mimics significantly suppressed luciferase activity from LOC146880-WT, but did not inhibit luciferase activity from LOC146880-MUT (Figure 3F). This suggested direct binding between LOC146880 and miR-328-5p. We then analyzed the expression levels of miR-328-5p in ESCC tissues and cell lines. MiR-328-5p expression levels were significantly reduced in ESCC tissues (TCGA-ESCC dataset and 21 paired ESCC tissues from Nanjing hospital) compared to adjacent normal esophageal tissues (Figure 3G, 3H). We observed inverse correlation between LOC146880 and miR-328-5p expression levels in ESCC and adjacent normal esophageal tissue samples (*n* = 21; R = –0.5542, *P* = 0.009; Figure 3I). Moreover, LOC146880 knockdown increased the expression levels of miR-328-5p in both Kyse30 and TE-1 cells (Figure 3J). These results suggested that LOC146880 promoted ESCC progression by sponging miR-328-5p.

### MiR-328-5p plays a tumor suppressor role in ESCC cells

**Figure 4:**
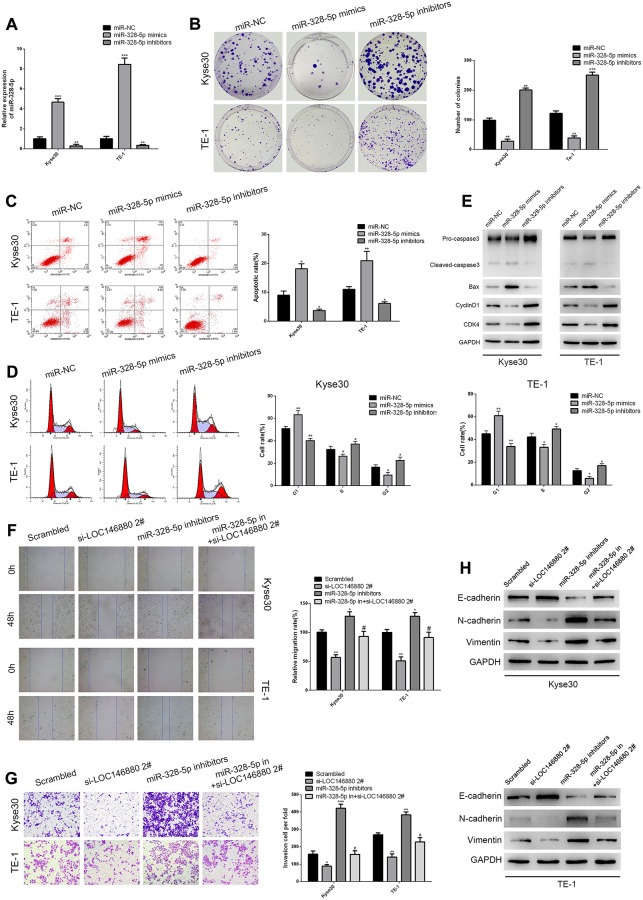
**MiR-328-5p functions as a tumor suppressor in ESCC cells.** (**A**) QRT-PCR analysis shows the expression levels of miR-328-5p in Kyse30 and TE-1 cells transfected with negative control (miR-NC), miR-328-5p mimics, and miR-328-5p inhibitors. (**B**) Colony formation assay results show the viability of ESCC cells transfected with miR-328-5p mimics and miR-328-5p inhibitors. (**C**) Flow cytometry analysis shows the apoptotic rate of ESCC cells transfected with miR-328-5p mimics and miR-328-5p inhibitors. (**D**) Flow cytometry analysis shows cell cycle distribution of ESCC cells transfected with miR-328-5p mimics and miR-328-5p inhibitors. (**E**) Western blot analysis demonstrates the expression levels of pro-apoptotic (cleaved caspase 3 and Bax) and cell cycle-related proteins (CDK4 and cyclinD1) in ESCC cells transfected with miR-328-5p mimics and miR-328-5p inhibitors. (**F**–**G**) wound healing and Transwell invasion assays show the (F) migration and (G) invasiveness of ESCC cells transfected with scrambled, si-LOC146880#2, miR-328-5p inhibitors, miR-328-5p inhibitors plus si-LOC146880#. (**H**) Western blot analysis shows the expression levels of EMT-related proteins, namely, E-cadherin, N-cadherin, and vimentin in ESCC cells transfected with scrambled, si-LOC146880#2, miR-328-5p inhibitors, miR-328-5p inhibitors plus si-LOC146880#.

QRT-PCR analysis confirmed that miR-328-5p levels were significantly upregulated in Kyse30 and TE-1 cells transfected with miR-328-5p mimics and significantly reduced in Kyse30 and TE-1 cells transfected with miR-328-5p inhibitors (Figure 4A). Colony formation ability was significantly reduced in miR-328-5p mimic-transfected ESCC cells and significantly increased in miR-328-5p inhibitor-transfected ESCC cells (Figure 4B). Flow cytometry analysis showed that overexpression of miR-328-5p significantly increased apoptosis and G1-phase cell cycle arrest of ESCC cells (Figure 4C, 4D). MiR-328-5p mimics decreased the levels of cell cycle proteins, cyclin-D1 and CDK4, and increased the levels of pro-apoptotic proteins (cleaved Caspase3 and Bax) in ESCC cells, whereas, miR-328-5p inhibitors reversed these effects (Figure 4C–4E). MiR-328-5p over-expression inhibited the migration and invasiveness of Kyse30 and TE-1 cells, whereas, miR-328-5p inhibitors reversed these effects (Figure 4F, 4G). Furthermore, Kyse30 and TE-1 cells co-transfected with miR-328-5p inhibitors and si-LOC146880#2 showed significantly reduced migration and invasiveness compared to those transfected with miR-328-5p inhibitors alone (Figure 4F, 4G). Kyse30 and TE-1 cells transfected with miR-328-5p inhibitors also showed reduced E-cadherin and increased N-cadherin and Vimentin expression levels, but these effects were reversed by LOC146880 knockdown (Figure 4H). These results demonstrated that miR-328-5p knockdown promoted EMT of ESCC cells via LOC146880.

### FSCN1 is the target gene of miR-328-5p in ESCC

**Figure 5:**
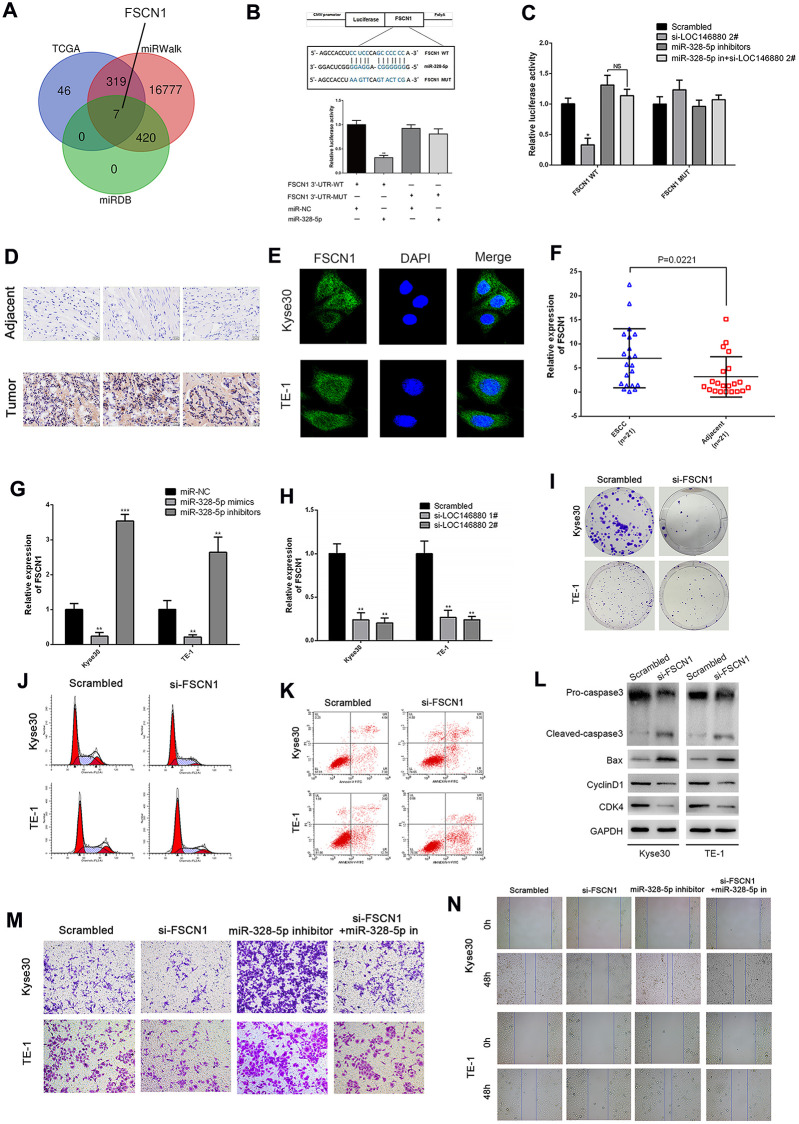
**FSCN1 is a direct target of miR-328-5p and enhances the progression of ESCC cells.** (**A**) TCGA, miRDB and miRWalk database analyses show that FSCN1 is a potential miR-328-5p target gene (FGF19, EN1, ADAM8, ELFN2, FSCN1, CLDN6, IFIT1). (**B**) The predicted miR-328-5p seed region in the wild-type (WT) and mutated (Mut) 3′UTR of FSCN1 is shown in the top panel. Dual luciferase reporter assay results (bottom panel) show relative luciferase activity of Kyse30 cells co-transfected with luciferase reporter plasmids containing wild-type or mutated 3′UTR of FSCN1 (WT or Mut) and miR-328-5p mimics, miR-NC act as control. (**C**) Dual luciferase reporter assay results show luciferase activity in control or LOC146880-silenced Kyse30 cells transfected with luciferase reporter vector with wild-type or mutated 3′UTR of FSCN1 plus miR-NC or miR-328-5p inhibitors. (**D**) IHC assay shows higher FSCN1 expression level in tumor tissues than in adjacent tissues. (**E**) ICC assay shows localization of FSCN1 in the cytoplasm and nucleus of ESCC cells. (**F**–**H**) QRT-PCR analysis shows expression levels of FSCN1 transcripts in (F) 21 pairs of esophageal cancer and adjacent normal esophageal tissues, (G) ESCC cells transfected with miR-NC, miR-328-5p mimics, and miR-328-5p inhibitors, and (H) control and LOC146880-silenced ESCC cells. (**I**) Colony formation assay results show the viability of si-NC and si-FCSN1-transfected Kyse30 and TE-1 cells. (**J**–**K**) Flow cytometry analysis shows (J) apoptotic rate and (K) cell cycle distribution of si-NC and si-FCSN1-transfected Kyse30 and TE-1 cells. (**L**) Western blot analysis shows the levels of apoptosis-related proteins (cleaved caspase 3 and Bax) and cell cycle proteins (cyclinD1 and CDK4) in si-NC and si-FCSN1-transfected Kyse30 and TE-1 cells. (**M**–**N**) Wound-healing and Transwell invasion assay results show (M) migration and (N) invasiveness of si-NC and si-FCSN1-transfected Kyse30 and TE-1 cells. ^*^P < 0.05, ^**^P < 0.01, ^***^P < 0.001.

Next, we searched TCGA, miRDB, and miRWalk databases and identified 7 potential target genes of miR-328-5p including FSCN1 (Figure 5A). We focused on the function of FSCN1 in ESCC cells. Fascin 1 (FSCN1) promotes progression of bladder cancer and non-small cell lung cancer cells [24, 25]. Dual luciferase reporter assays showed that miR-328-5p mimics significantly reduced luciferase activity in Kyse30 cells co-transfected with luciferase vector with wild-type 3′UTR of FSCN1 (FSCN1-WT), but did not affect luciferase activity in Kyse30 cells co-transfected with luciferase vector with mutated 3′UTR of FSCN1 (FSCN1-MUT) (Figure 5B). Moreover, luciferase activity was significantly reduced in LOC146880-silenced Kyse30 cells transfected with the FSCN1-WT luciferase vector, but these effects were reversed by co-transfecting LOC146880-silenced Kyse30 cells with miR-328-5p inhibitors (Figure 5C).

We then performed IHC assays to compare expression of FSCN1 in ESCC and adjacent normal esophageal tissues. As expected, FSCN1 protein level was higher in tumor tissues than in adjacent tissues (Figure 5D). Immunocytochemistry (ICC) assays showed that FSCN1 protein levels were significantly higher in the cytoplasm than the nucleus in cells within ESCC tissues (Figure 5E). QRT-PCR analysis showed that FSCN1 expression levels were significantly higher in ESCC tissues compared to adjacent normal esophageal tissues (Figure 5F). We observed negative correlation between FSCN1 and miR-328-5p expression levels in ESCC tissues (Supplementary Figure 2A). In contrast, FSCN1 expression positively correlated with LOC146880 expression levels in ESCC tissues (Supplementary Figure 2B). Moreover, inhibition of miR-328-5p significantly upregulated FSCN1 levels, whereas, miR-328-5p overexpression suppressed FSCN1 levels in ESCC cells (Figure 5G). Furthermore, knockdown of LOC146880 down-regulated the expression of FSCN1 in ESCC cells (Figure 5H).

FSCN1 knockdown significantly reduced colony formation ability of ESCC cells compared to the controls (Figure 5I and Supplementary Figure 2C). Flow cytometric analysis showed that silencing of FSCN1 increased apoptosis and G0/G1 phase cell cycle arrest of ESCC cells (Figure 5J, 5K and Supplementary Figure 2D, 2E). Western blot analysis showed that FSCN1 silencing decreased pro-caspase3, cyclin D1, and CDK4 protein levels, and increased cleaved caspase3 and Bax protein levels (Figure 5L). Furthermore, silencing of FSCN1 suppressed invasiveness and migration ability of Kyse30 and TE-1 cells co-transfected with miR-328-5p inhibitors compared to the corresponding controls (Figure 5M, 5N and Supplementary Figure 2F, 2G).

### LOC146880/miR-328-5p/FSCN1 axis promotes ESCC progression via MAPK signaling pathway

**Figure 6:**
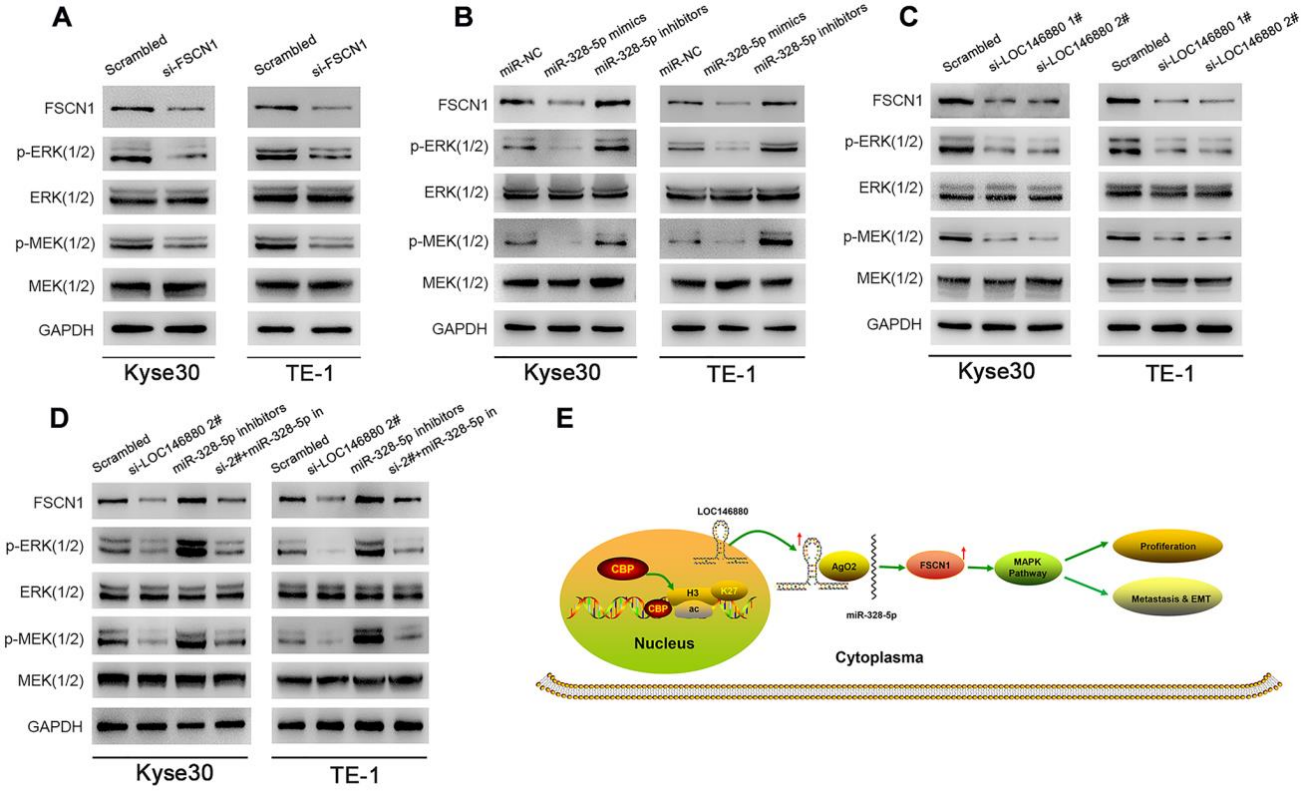
**FSCN1 regulates ESCC cell growth and progression via MAPK signaling pathway in a LOC146880-dependent manner.** (**A**–**D**) Western blotting analysis shows levels of FSCN1 and MAPK signaling pathway proteins, MEK (1/2), ERK (1/2), p-MEK (1/2) and p-ERK (1/2) in (A) Kyse30, TE-1 transfected with control and si-FSCN1, (B) Kyse30 and TE-1 cells transfected with miR-NC, miR-328-5p mimics and miR-328-5p inhibitors, and (C) control and LOC146880-silenced ESCC cells, (D) Kyse30 and TE-1 cells transfected with scrambled, si-LOC146880#2, miR-328-5p inhibitors, miR-328-5p inhibitors plus si-LOC146880#. (**E**) Schematic representation shows that H3K27 acetylation-induced LOC146880 over-expression promotes ESCC growth and progression by upregulating FSCN1/MAPK signaling pathway via sponging of miR-328-5p.

Previous studies showed that FSCN1 promoted breast cancer and non-small cell lung cancer progression through the MAPK pathway [25, 26]. Therefore, we analyzed MEK1/2, ERK1/2, phosphorylated MEK1/2 and phosphorylated ERK1/2 levels in ESCC cells. Reduced expression of FSCN1 decreased FSCN1, phospho-MEK1/2, and phospho-ERK1/2 levels in ESCC cells (Figure 6A). Furthermore, overexpression of miR-328-5p significantly decreased FSCN1, phospho-MEK1/2, and phospho-ERK1/2 levels in ESCC cells, but these effects were reversed by miR-328-5p inhibitors (Figure 6B). Moreover, LOC146880 silencing downregulated the expression of FSCN1, phospho-MEK1/2, and phospho-ERK1/2 levels in ESCC cells (Figure 6C), but these effects were reversed by miR-328-5p inhibitors (Figure 6D). Taken together, these data demonstrated that LOC146880 enhanced growth and progression of ESCC by sponging miR-328-5p and upregulating FSCN1 and MAPK signaling pathway (Figure 6E).

### LOC146880 knockdown inhibits *in vivo* ESCC growth and progression

**Figure 7:**
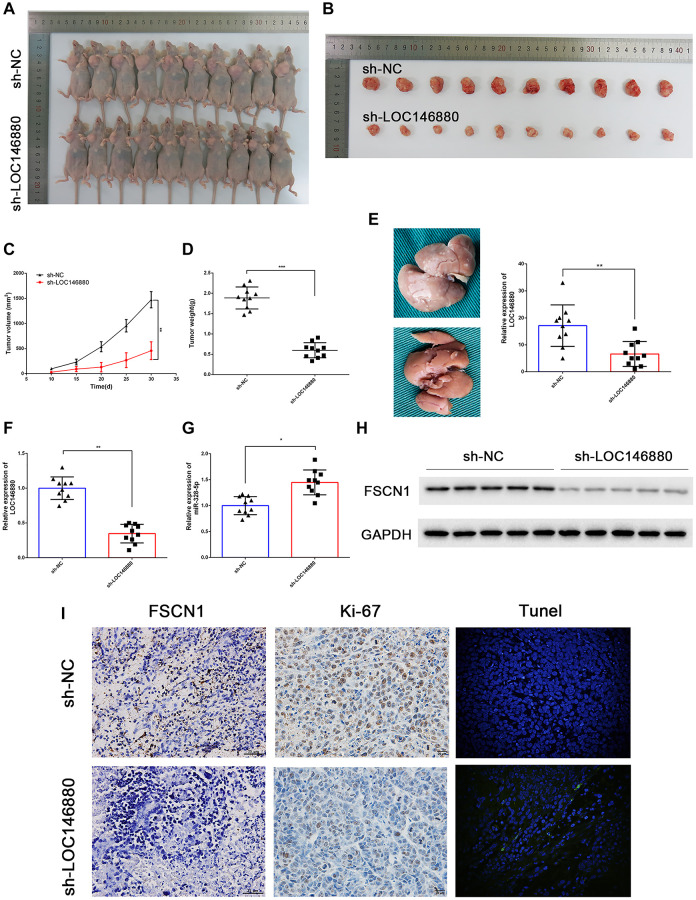
**LOC146880 silencing reduces *in vivo* ESCC growth and progression.** (**A**) Representative images show nude mice xenografted with sh-NC-transfected Kyse30 cells (upper, *n* = 10) and sh-LOC146880-transfected Kyse30 cells (lower, *n* = 10). (**B**) Representative images show ESCC-derived tumor xenografts from nude mice injected with sh-NC-transfected Kyse30 cells (upper) and sh-LOC146880-transfected Kyse30 cells (lower). (**C**) Tumor volumes of sh-NC and sh-LOC146880 groups of ESCC xenograft tumors in nude mice. (**D**) Tumor mass of sh-NC and sh-LOC146880 groups of ESCC xenograft tumors in nude mice. (**E**) Representative images and quantitative analysis show the number of metastatic tumor nodules in the liver of nude mice belonging to sh-NC and sh-LOC146880 groups. (**F**–**G**) QRT-PCR analysis of (F) LOC146880 and (G) miR-328-5p expression levels in xenograft tumors from sh-NC and sh-LOC146880 groups. (**H**) Western blot analysis shows FSCN1 protein levels in xenograft tumors from sh-NC and sh-LOC146880 groups. (**I**) Immunohistochemical staining and Tunel Staining show3 expression levels of FSCN1, Ki-67 and apoptotic cells in xenograft tumor sections from sh-NC and sh-LOC146880 groups.

Next, we analyzed the *in vivo* growth and progression of xenografted sh-NC- and sh-LOC146880-transfected ESCC cells. We injected twenty male BALB/c nude mice with sh-NC or sh-LOC146880-transfected Kyse30 cells. The sh-LOC146880 group showed significant reduction in tumor volume and tumor mass compared to the sh-NC group (Figure 7A–7D). We then generated the liver metastasis model mice by injecting sh-NC- or sh-LOC146880-transfected Kyse30 cells under the splenic capsule of the nude mice. The number of visible metastatic tumor nodules on the liver surface were significantly reduced in the sh-LOC146880 group compared to the sh-NC group (Figure 7E). Furthermore, LOC146880 and FSCN1 expression levels were significantly reduced and miR-328-5p levels were significantly increased in the xenograft tumors derived from the sh-LOC146880 group compared to those from the sh-NC group (Figure 7F–7H). Immunohistochemical staining demonstrated reduced expression of FSCN1 and Ki-67 proteins in the xenograft tumors derived from the sh-LOC146880 group compared to the sh-NC group (Figure 7I). Furthermore, IHC results showed significantly higher numbers of cleaved caspase3-positive cells in the xenograft tumors derived from the sh-LOC146880 group compared to the control group (Figure 7I). These results confirmed that LOC146880 promoted *in vivo* ESCC growth and progression.

## DISCUSSION

Pseudogene-derived lncRNAs play a critical role in malignant tumors. LncRNA LOC146880 promotes lung cancer, but suppresses growth and progression of gastric cancer cells [21, 22]. In the current study, we demonstrated that LOC146880 promoted ESCC progression by activating the MAPK signaling pathway via miR-328-5p/FSCN1 axis.

Our study demonstrated that LOC146880 was significantly overexpressed in ESCC tissues compared to the adjacent non-tumor esophageal tissues. Furthermore, higher expression of LOC146880 correlated with worse prognosis of ESCC patients. LOC146880 knockdown suppressed *in vitro* proliferation, migration and invasion of ESCC cells. Moreover, LOC146880 knockdown suppressed EMT and increased apoptosis as well as G1 phase cell cycle arrest of ESCC cells. LOC146880 knockdown increased the expression of pro-apoptotic proteins, cleaved caspase-3 and Bax. *In vivo* tumor growth and metastasis of xenografted LOC146880 knockdown ESCC cells was significantly lower compared to sh-NC-transfected ESCC cells. These results demonstrated that lncRNA LOC146880 acted as an oncogene in ESCC. Overall, LOC146880 knockdown suppressed *in vitro* and *in vivo* ESCC growth and progression via miR-328-5p/FSCN1/MAPK axis.

LncRNAs act as ceRNAs for specific miRNAs, thereby regulating the expression of miRNA-targeted genes [27–29]. In this study, bioinformatics analysis, dual luciferase reporter assays, and RIP assays demonstrated that miR-328-5p was the target miRNA for LOC146880. MiR-328-5p plays an oncogenic role in hepatocellular carcinoma, lung cancer, and breast cancer [30–32]. However, miR-328 functions as a tumor suppressor in cervical cancer, gastric cancer, and nasopharyngeal carcinoma [33–35]. Moreover, overexpression of miR-328 inhibited proliferation and invasion of EC109 and EC9706 esophageal cancer cells [36]. In this study, we demonstrated that miR-328-5p overexpression induced G1 phase cell cycle arrest and suppressed proliferation, invasion, migration, and EMT of ESCC cells. However, these effects were reversed by miR-328 knockdown. Moreover, increased proliferation, invasion, migration, and EMT of miR-328-5p inhibitor-transfected ESCC cells were reversed by LOC146880 knockdown.

Bioinformatics analysis and dual luciferase reporter assay results confirmed that FSCN1 was the target gene of miR-328-5p. FSCN1 is a member of the fascin family of actin-binding proteins, and regulates cellular migration, motility, adhesion, and inter-cellular interactions [37]. The oncogenic role of FSCN1 has been described in multiple cancers including ESCC [24, 38–40]. FSCN1 is targeted by multiple lncRNA-miRNA pairs in ESCC such as PVT1-miR-145, TTN-AS1-miR-133, and ROR-miR-145 [41–43]. Our study demonstrated that FSCN1 was up-regulated in ESCC tissues and its knockdown significantly reduced proliferation, invasion and migration of ESCC cells. Knockdown of LOC146880 significantly suppressed FSCN1 expression, whereas, inhibition of miR-328-5p up-regulated the expression of FSCN1 in ESCC cells. MiR-328-5p silencing reversed the effects of FSCN1 knockdown and increased the invasion and migration of ESCC cells. Furthermore, knockdown of LOC146880 reduced the levels of p-ERK and p-MEK, key members of the MAPK signaling pathway. In contrast, miR-328-5p silencing increased p-ERK and p-MEK levels. Moreover, miR-328-5p inhibitors enhanced MAPK pathway activation in LOC146880-silenced ESCC cells. These findings suggested that LOC146880 enhanced ESCC progression by sponging miR-328-5p, thereby upregulating FSCN1 and MAPK signaling pathway activation.

Previous studies have shown that expression of pseudogene-derived lncRNAs is regulated mainly by epigenetic mechanisms [44, 45]. In this study, we demonstrated that acetylated histone H3 (H3K27ac) levels were upregulated in the LOC146880 promoter. ChIP assay analysis confirmed that H3K27ac levels were significantly higher in the LOC146880 promoter region of ESCC cells and tissues compared to their corresponding controls. We further analyzed the expression of CBP, a key regulator of chromatin acetylation, in the promoter region of LOC146880. As expected, CBP levels were significantly enriched in the LOC146880 promoter region of both KYSE30 and TE-1 cells compared to HEEC cells. Moreover, the expression of LOC146880 was significantly reduced by CBP silencing. These results suggested that LOC146880 expression was induced by increased CBP and histone acetylation in its promoter region in the ESCC cells and tissues.

In conclusion, our study demonstrated that pseudogene-derived lncRNA LOC146880 promoted *in vitro* and *in vivo* growth and progression of ESCC cells by increasing the expression of FSCN1 and MAPK signaling pathway activation via sponging of miR-328-5p. Therefore, our study shows that LOC146880-miR-328-5p-FSCN1 axis is a promising therapeutic target in ESCC.

## MATERIALS AND METHODS

### ESCC tissue samples

In this study, 21 ESCC tissues were collected from ESCC patients that underwent esophagectomy from 2016 to 2018 at the First Affiliated Hospital of Nanjing Medical University. The adjacent non-tumor tissues from each ESCC patients were collected act as control. All patients were diagnosed with ESCC based on histopathological evaluation and did not receive chemotherapy or radiotherapy before esophagectomy. All tissue samples were frozen in liquid nitrogen immediately and then stored at –80°C for further analyses. This study was approved by the Research Ethics Committee of Nanjing Medical University. We obtained written informed consent from all patients before conducting the study.

### Cell culture and transfections

We purchased six human ESCC cell lines (ECA109, TE-1, Kyse-30, Kyse-70, Kyse-150 and Kyse-410) and normal human esophageal epithelial cell line (HEEC) from the Cell Bank of Chinese Academy of Sciences (Shanghai, China). The cells were cultured in RPMI-1640 medium supplemented with 10% fetal bovine serum (FBS), 100 units of penicillin/mL and 100 ng of streptomycin/mL at 37^o^C and 5% CO_2_. We purchased 3 LOC146880-specific si-RNAs (si-LOC146880 #1, si-LOC146880 #2, and si-LOC146880#3), miR-328-5p mimics, miR-328-5p inhibitors, si-FSCN1, and corresponding non-specific controls from Invitrogen, USA. We also purchased short hairpin RNA (shRNAs) against LOC146880 (sh-LOC146880) from Sangon Biotech Co. Ltd. (Shanghai, China).

### Quantitative RT-PCR

Total RNA was isolated from tissues or cells using Trizol (Invitrogen, USA) according to manufacturer’s recommendations. Then, RNA (1 μg) samples were reverse transcribed into complementary DNA (cDNA) using the Prime Script Reverse Transcription Kit (Takara Bio, Inc., Otsu, Japan). We then designed and purchased Bulge-loop™ miRNA qRT-PCR primers (one RT primer and a pair of qRT-PCR primers for each set) specific for miR-328-5p from RiboBio (Guangzhou, China). Quantitative polymerase chain reaction (qPCR) was performed using gene-specific primer sets and SYBR Premix Ex Taq (Takara Bio, Inc.) in an ABI StepOne Plus system (Applied Biosystems, CA, USA). The reaction conditions were 1 cycle of 95°C for 30 s followed by 45 cycles of 95°C for 5 s and 60°C for 30 s. All samples were analyzed in triplicate. Each experiment was repeated three times. The expression levels of target genes relative to β-actin were determined relative to β-actin by the 2^−ΔΔCt^ method. The expression levels of miRNAs were normalized to U6. The qPCR primer sequences used in this study are shown in Supplementary Table 1.

### Protein extraction and western blotting

Total protein extracts from ESCC tissue samples and cultured ESCC cells were prepared with ice-cold RIPA buffer containing protease inhibitors. The cleared protein lysates were obtained by centrifugation at 10,000 rpm for 15 min at 4°C and quantified using BCA Protein Quantitation Assay (KeyGEN, China) with bovine serum albumin (BSA) as standard. The protein samples (30 μg) were separated by SDS polyacrylamide gel electrophoresis (SDS-PAGE), transferred to polyvinylidene fluoride (PVDF) membranes, and blocked using 5% skimmed milk for 30 mins. Then, the membranes were incubated overnight at 4^o^C with specific primary antibodies against CDK4 (Abcam; ab137675; 1:1000), cyclinD1 (Abcam; ab226977; 1:2000), Caspase-3 (Cell Signaling Technology; 9662S; 1:1000), cleaved Caspase-3 (Cell Signaling Technology; 9664S; 1:2000), Bax (Abcam; ab53154; 1:1000), E-cadherin (Abcam; ab15148; 1:2000), N-cadherin (Abcam; ab18203; 1:2000), Vimentin (Abcam; ab137321; 1:1000), and mouse anti-β-actin antibody (Abcam; ab8227; 1:5000). Then, the blots were incubated with HRP-conjugated anti-rabbit IgG antibody (Abcam; ab205718; 1:20000) or HRP-conjugated anti-mouse IgG antibody (Abcam; ab205719; 1:2000) for 1 h at room temperature. The blots were then developed with enhanced chemiluminescence (ECL; Cell Signaling Technology). The density of the protein bands was quantified using Image J software version 1.42q (National Institutes of Health, Bethesda, Maryland, USA).

### RNA immunoprecipitation

We performed RNA immunoprecipitation (RIP) assay with anti-Ago2 antibodies using the Magna RIP™ RNA-Binding Protein Immunoprecipitation Kit (Millipore, Billerica, MA, USA). Briefly, cells were incubated with the RIP lysis buffer for 30 min. Then, the crude cellular lysate was incubated at 4°C for 60 min with protein A magnetic beads conjugated with anti-Ago2 antibodies (Abcam; Cat. No. ab32381) or normal mouse IgG (Abcam). The levels of miRNAs and LOC146880 in the immunoprecipitated RNA were analyzed by qRT-PCR. Total RNA was used as input control.

### Colony formation assay

ESCC cells (1000 cells/well) in RPMI-1640 containing 10% FBS were cultured in a six-well plate in an humidified incubator at 5% CO_2_ and 37°C for 2 weeks. The medium was changed every third day. After two weeks, the colonies were fixed in methanol for 30 min and stained with 0.1% crystal violet (Sigma-Aldrich, USA) for 15 mins. The number of colonies (50 cells or more) in each experimental group were counted under a light microscope (Olympus, Tokyo, Japan).

### Immunofluorescence

Immunofluorescence staining of ESCC cells with anti-Ki67 and anti-FSCN1 antibodies was performed as described previously [46]. Briefly, cells were washed thrice in cold 0.01M PBS, and fixed in 4% paraformaldehyde for 30 min at room temperature. The cells were then incubated with immunostaining blocking buffer containing 0.25% Triton X-100 (Beyotime, Shanghai, China) for 10 min, washed thrice with PBS, and blocked with 5% BSA at room temperature for 1 h. Subsequently, cells were incubated overnight at 4°C with rabbit anti-Ki67 antibody (1:100; ab15580; Abcam, Cambridge, MA, USA) followed by incubation with Alexa Fluor 488-conjugated anti-rabbit secondary antibody (Abcam; 1:500; ab150077) for 1h at room temperature. The cells were counterstained with DAPI (nuclear dye) at room temperature for 15 min. Finally, all samples were photographed and analyzed using a Leica TCS SP8-MaiTai MP confocal laser scanning microscope (Leica, Wetzlar, Germany).

### Transwell invasion assay

Transwell invasion assay was performed in Boyden chambers (BD Bio-sciences). We seeded 4 × 10^4^ ESCC or HEEC cells in serum-free RPMI-1640 medium per well in the top chamber and RPMI-1640 medium with 10% FBS in the bottom chamber. The chambers were incubated at 37°C and 5%CO_2_. After 24 h, the cells in the top chamber were removed with a cotton swab. The cells in the membrane were fixed in 4% paraformaldehyde for 15 min followed by staining with 0.1% crystal violet (Beyotime, China) for 15 min. The stained cells in each well were counted in five random fields and photographed under a light microscope.

### Wound healing assay

ESCC or HEEC cells were seeded in a six-well plate at a density of 5 × 10^4^ cells/well and cultured until they reached 80–90% confluency. Then, the cellular monolayer was wounded by scratching with a P200 pipette tip. The non-adherent cells were removed by washing with culture medium. Then, the cells were incubated at 37°C for 48 h and photographed to identify the gap area.

### Dual luciferase reporter assay

We cloned wild type or mutated LOC146880 and 3′-untranslated regions (3′-UTR) of FSCN1 into the pGL3-basic luciferase reporter vector (Promega,USA). Then, we co-transfected ESCC cells with 400 ng of recombinant pGL3 vectors for LOC146880 (pGL3-LOC146880-WT, pGL3-LOC146880-WT, or empty pGL3 empty vector) or recombinant pGL3 vectors for FSCN1 (pGL3-FSCN1-WT, pGL3-FSCN1-MUT, or empty pGL3 vector) plus 40 ng of pRL-TK plasmid (Renilla luciferase control vector) plus 50 nM miR-4715-3p mimic, miR-328-5p mimic, miR-4492 mimic or corresponding negative controls (miR-NC). After 48 h, the cells were harvested and firefly and Renilla luciferase activities were estimated using dual-luciferase reporter assay system (Promega, USA) according to the manufacturer’s protocol. Renilla luciferase activity was used to normalize transfection efficiency.

### Flow cytometry

For analyzing cellular apoptosis, ESCC cells (Kyse30 and Te-1) were first trypsinized for 5 min and then incubated in the dark for 20 min at room temperature with staining buffer containing 5μL of 7-AAD and 5μL of APC-conjugated Annexin V. The stained cells were analyzed in a FACScan flow cytometer (BD Biosciences, Franklin Lakes, USA). The percentage of apoptotic cells treated with Annexin-V FITC/PI in all experimental groups were calculated using the ModFit 3.0 software (Verity Software House, Topsham, ME, USA).

For cell cycle analysis, logarithmically growing ESCC and HEEC cells were fixed overnight with cold 70% alcohol. Then, they were incubated at room temperature for 30 min with 50mg/L RNaseA, and stained in the dark with propidium iodide (BD Biosciences, Franklin, NJ, USA) for 30 min at 37°C. The stained cells were analyzed using a FACScan flow cytometer (BD Biosciences, NJ, USA) and the percentage of G1, S, and G2-M cells were calculated using the ModFit 3.0 software (Verity Software House, Topsham, ME, USA).

### Fluorescence *in situ* hybridization (FISH)

We fixed Kyse30 and Te-1 cells in 4% formaldehyde. The fixed ESCC cells were then incubated overnight at 4°C with hybridization buffer containing the 0.3–0.6 μM LOC-146880 probe labeled by Cy3(Sangon Biotech). Then, the probe signal was developed using the Fluorescent *In Situ* Hybridization Kit (GenePharma, China) according to the manufacturer’s instructions. The nuclei were stained with DAPI (Sigma) for 10 min. The images were captured with the Nikon Ni-U fluorescence microscope (Nikon, Japan).

### Animal experiments

Twenty male Balb/c nude mice (4–6 weeks old) were obtained from the Shanghai Laboratory Animal Center (Chinese Academy of Sciences, Shanghai, China), and maintained in a pathogen-free, temperature- and humidity-controlled environment (25 ± 2°C, 50 ± 5% humidity). The animal experiments were performed in accordance to the Animal Management Rule (Document 55, 2001) of the Chinese Ministry of Health and the protocols approved by the Ethics Committee of Nanjing University.

For the subcutaneous xenograft tumor experiments, the nude mice were injected subcutaneously with Kyse30 cells (3 × 10^6^) stably transduced with sh-NC or sh-LOC146880 (*n* = 10 per group) into either side of the axillary area. The length (L) and width (W) of the tumors were measured once a week. The tumor volume was estimated as V = 0.5 × L × W^2^. The xenograft tumor-laden nude mice were euthanized after four weeks and the subcutaneous tumors were photographed. The tumor tissues were cut and preserved at −80°C for further experiments.

For *in vivo* metastasis experiments, four-week-old BALB/c nude mice were randomly divided into two experimental groups (*n* = 10 for each group). After anesthesia, the mouse spleen was surgically exposed and 5 × 10^6^ sh-LOC1466880 or sh-NC-transfected Kyse30 cells were slowly injected under the splenic capsule. Five minutes after injection, the spleen was removed. Two months later, the mice were sacrificed and the livers were initially examined by the naked eye. The metastatic colonies on the liver were visually counted in both groups.

### Immunohistochemistry (IHC)

The murine xenograft tumor specimens were stained with antibodies against FSCN1 (Abcam; ab126772; 1:500), Ki-67 (Abcam; ab15580; 1:200) and Cleaved Caspase-3 (Abcam; ab2302; 1:20) and the staining intensity was measured in both groups. Immunohistochemical staining of 4 μm-thick TMA slides with ESCC and adjacent normal esophageal tissue samples was performed as previously described [47].

### 3D spheroidal cell migration assay

ESCC cells (5 × 10^3^ cells per well) were seeded in low-adherence round-bottom 96-well plates (Nunc). After 2 days of spheroid formation, the supernatant was replaced with three-dimensional collagen I gel. To assist with collagen polymerization, we carefully added 1M sodium hydroxide (NaOH) to adjust the pH to 7.5. The images (4× magnification) were photographed at 0 and 48 h using the EVOS Cell Imaging System (Advanced Microscopy Group).

### Statistical analysis

The experimental data was analyzed using the GraphPad Prism Software 6.0 (GraphPad Software Inc., USA). The data between two groups were analyzed using paired or unpaired two-tailed Student’s *t* tests. A two-tailed Pearson’s correlation test was used to estimate association between LOC146880, miR-328-5p, and FSCN1 expression levels in ESCC and adjacent normal esophageal tissues. The data between multiple groups was analyzed using one-way ANOVA with Tukey’s post hoc test. Kaplan-Meier survival curve analysis was performed using the Kaplan-Meier Plotter database (https://kmplot.com/analysis/). All data are represented as means ± SEM. *P* < 0.05 was considered statistically significant.

### Availability of data and materials

The datasets from the current study are available from the corresponding author on reasonable request.

## Supplementary Materials

Supplementary Figures

Supplementary Table 1
