# Correction for: LncRNA LOC146880 promotes esophageal squamous cell carcinoma progression via miR-328-5p/FSCN1/MAPK axis

**DOI:** 10.18632/aging.203705

**Published:** 2021-12-14

**Authors:** Jianwei Tang, Honglei Xu, Qiang Liu, Jianan Zheng, Cheng Pan, Zhihua Li, Wei Wen, Jun Wang, Quan Zhu, Zhibo Wang, Liang Chen

**Affiliations:** 1 Department of Thoracic Surgery, The First Affiliated Hospital of Nanjing Medical University, Nanjing 210029, Jiangsu Province, China

**Keywords:** correction

Original article: Aging. 2021; 13:14198–14218.  . https://doi.org/10.18632/aging.203037

**This article has been corrected:** The authors recently found errors in **Figure 2**. The images for the Si-LOC146880 1# (Kyse30) and Si-LOC146880 2# (Kyse30) on top panel 2F had partial duplication. The images for the Scrambled (TE-1) and Si-LOC146880 1# (TE-1) on bottom panel 2F had partial duplication. The images Si-LOC146880 1# (Kyse30) and Si-LOC146880 1# (TE-1) in Figure 2G were the same image. The authors corrected panel 2F and 2G in Figure 2 by using representative images from the original sets of experiments. These alterations do not affect the results or conclusions of this work. The authors would like to apologize for any inconvenience caused.

New **Figure 2** is presented below.

**Figure 2:**
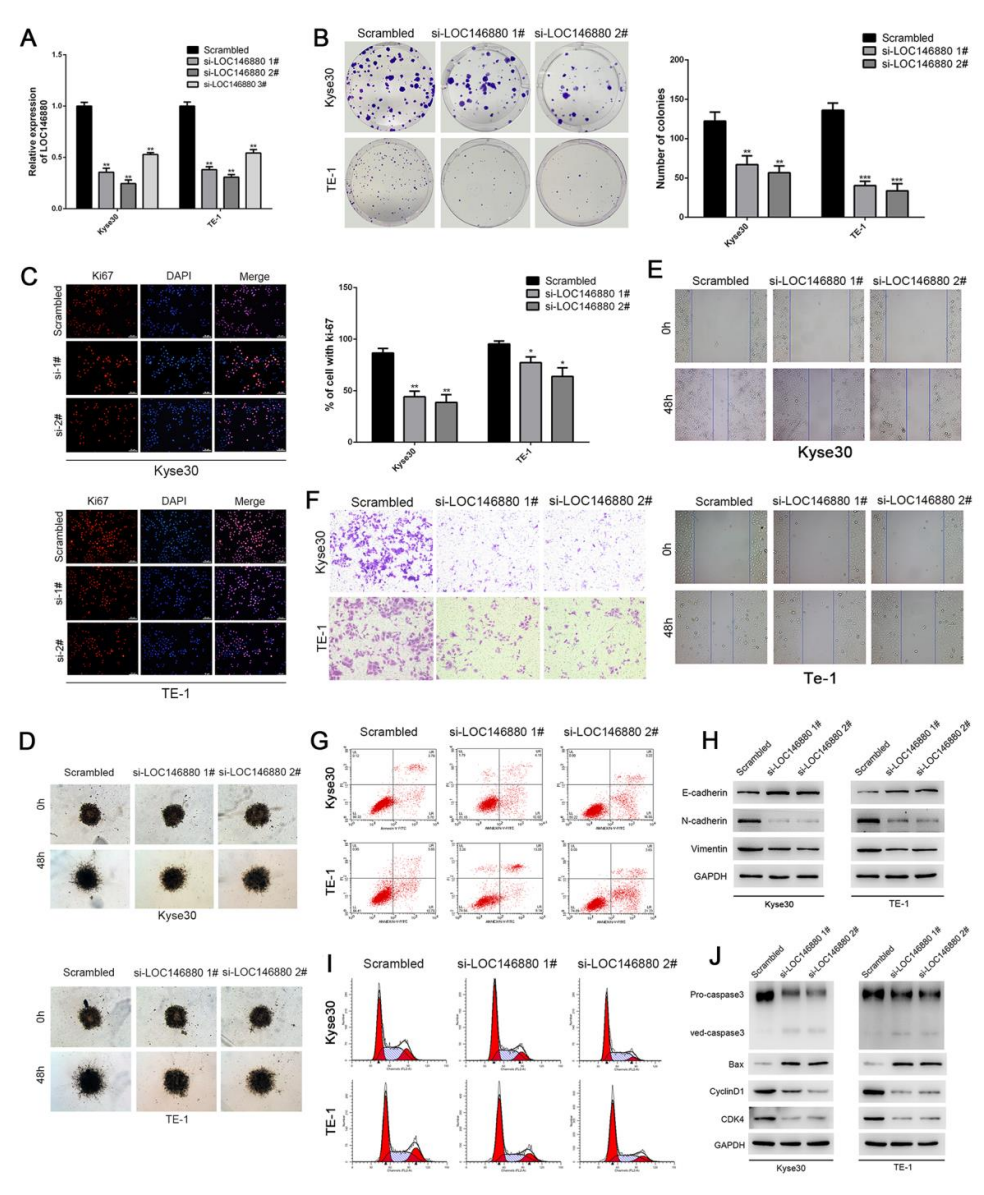
**Knockdown of LOC146880 inhibits growth and progression of ESCC cells. **(**A**) QRT-PCR analysis shows LOC146880 expression levels in ESCC cells transfected with si-NC (scrambled control siRNA), si-LOC146880#1, si-LOC146880#2, and si-LOC146880#3. (**B**) Colony formation assay results show viability of Kyse30 and TE-1 cells respectively transfected with si-NC, si-LOC146880#1, or si-LOC146880#2. (**C**) Immunofluorescence assay results show Ki-67 expression levels in control and LOC146880-silenced Kyse30 and TE-1 cells. (**D**) 3-dimensional spheroid assay results show the migration ability of control and LOC146880-silenced Kyse30 and TE-1 cells. (**E**) Wound healing assay results show the migration ability of control and LOC146880-silenced Kyse30 and TE-1 cells. (**F**) Transwell assay results show the invasiveness of control and LOC146880-silenced Kyse30 and TE-1 cells. (**G**) Flow cytometry analysis shows apoptotic rates of control and LOC146880-silenced Kyse30 and TE-1 cells. (**H**) Western blot analysis shows expression levels of E-cadherin (epithelial cell marker) as well as N-cadherin and vimentin (mesenchymal cell markers) in control and LOC146880-silenced Kyse30 and TE-1 cells. (**I**) Flow cytometry analysis shows cell cycle distribution of control and LOC146880-silenced Kyse30 and TE-1 cells. (**J**) Western blot analysis shows the levels of pro-apoptotic proteins (cleaved caspase-3 and Bax) and cell cycle proteins (cyclinD1 and CDK4) in control and LOC146880-silenced Kyse30 and TE-1 cells. *P < 0.05, **P < 0.01, ***P < 0.001.

