# Supplementary material for: LncRNA LOC146880 promotes esophageal squamous cell carcinoma progression via miR-328-5p/FSCN1/MAPK axis
Source: Aging (Albany NY). 2021 May 18;13(10):14198–218. doi: 10.18632/aging.203037 (PMC8202886; doi:10.18632/aging.203037)
Supplement: Supplementary Figures [file aging-13-203037-s001.pdf]

SUPPLEMENTARY FIGURES

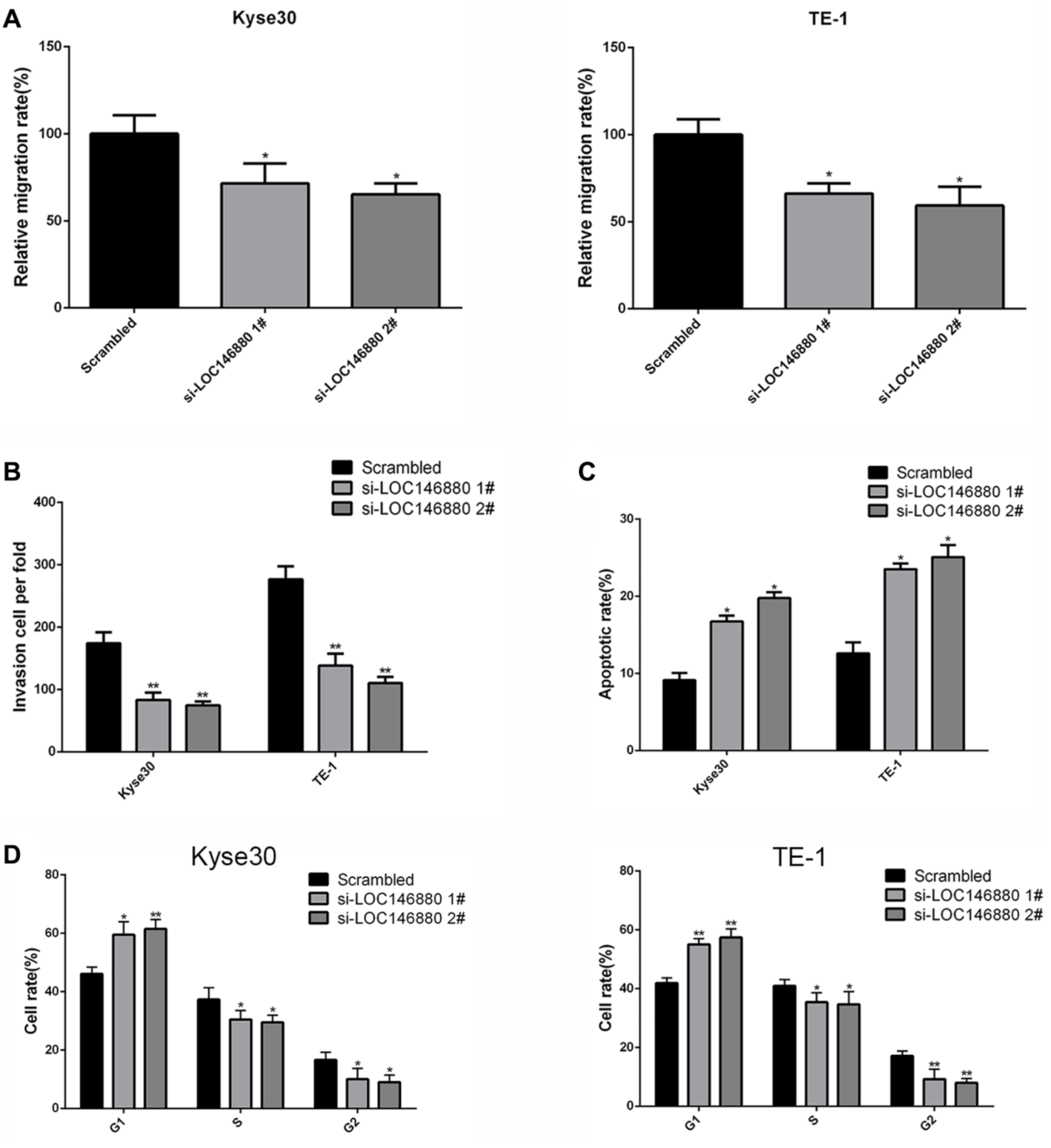

Supplementary Figure 1. Effects of LOC146880 silencing on migration, invasion, apoptosis and cell cycle distribution of ESCC cells. (**A**) Wound healing assay analysis of control and LOC146880 knockdown Kyse30 and Te-1 cells. (**B**) Transwell invasion assay analysis of control and LOC146880 knockdown Kyse30 and Te-1 cells. (**C–D**) Flow cytometry analyses of (C) apoptosis and (D) cell cycle distribution of control and LOC146880 knockdown Kyse30 and Te-1 cells.

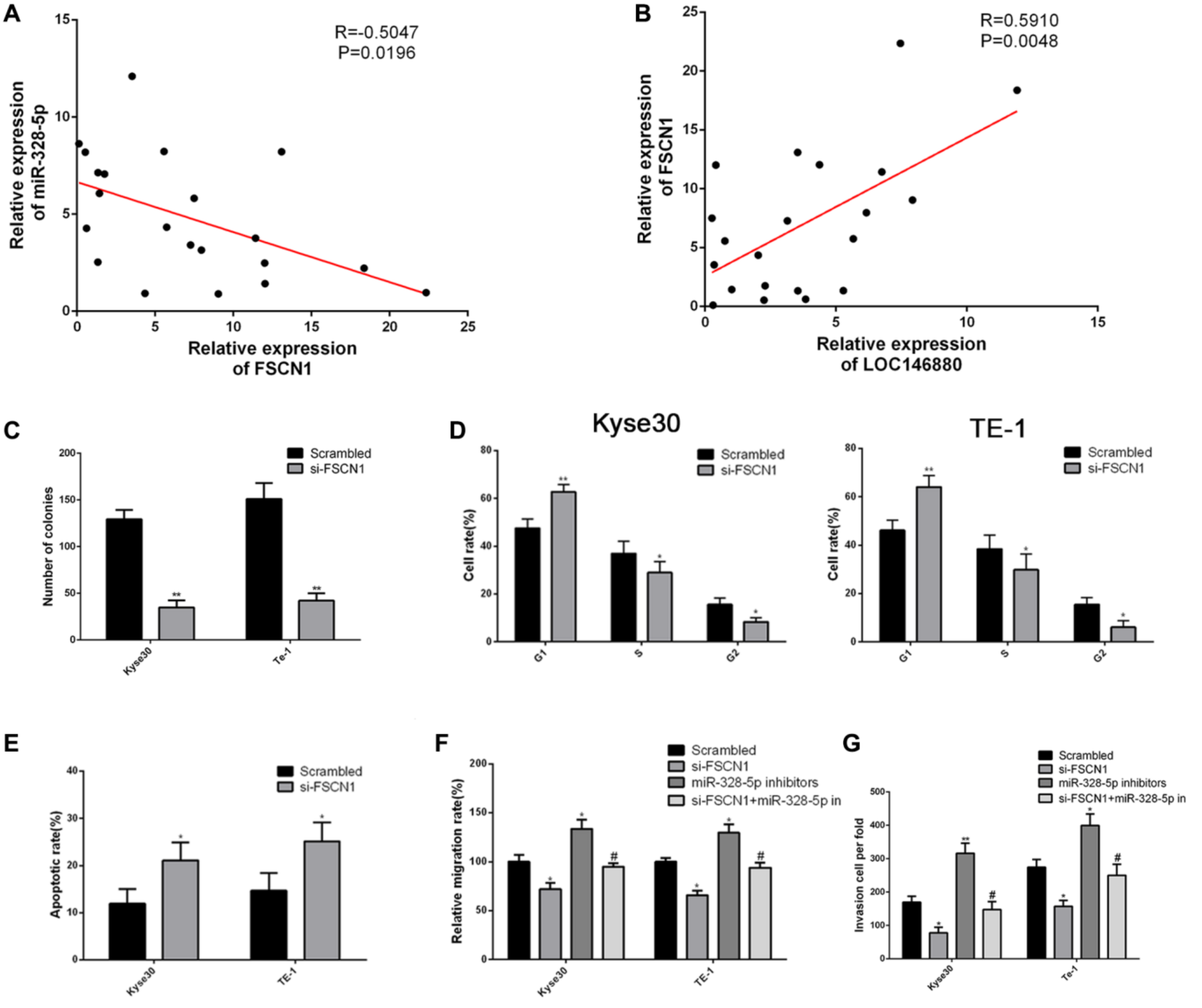

Supplementary Figure 2. FSCN1 expression correlates with miR-328-5p and LOC146880 expression levels in ESCC cells. (**A**–**B**) Spearman's correlation analysis shows (A) inverse relationship between FSCN1 and miR-328-5p expression levels, and (B) positive correlation between FSCN1 and LOC146880 expression levels in ESCC tissues. (**C**) Colony formation assay analysis of control and FSCN1-knockdown Kyse30 and TE-1 cells. (**D**–**E**) Flow cytometry analyses of apoptosis and cell cycle distribution of control and FSCN1-knockdown Kyse30 and TE-1 cells. (**F**–**G**) Wound healing and Transwell invasion assay analyses of control and FSCN1-knockdown Kyse30 and TE-1 cells co-transfected with or without miR-328-5p inhibitors.
