# Supplementary material for: LncRNA LOC146880 promotes esophageal squamous cell carcinoma progression via miR-328-5p/FSCN1/MAPK axis
Source: Aging (Albany NY). 2021 May 18;13(10):14198–218. doi: 10.18632/aging.203037 (PMC8202886; doi:10.18632/aging.203037)
Supplement: Supplementary Table 1 [file aging-13-203037-s002.pdf]

SUPPLEMENTARY TABLE

Supplementary Table 1. Information of the qPCR primer sequences and silencing RNA sequences.

qPCR primer name	Sequence (5′–3′)
LOC146880 (Forward)	AACTAAGGCACGGTCAACTATCA
LOC146880 (Reverse)	GGAAACTTTGGCTTTAAAACGA
miR-328-5p (Forward)	GGGGGGCAGGAGGGGC
miR-328-5p (Reverse)	AGTGCAGGGTCCGAGGTATT
FSCN1 (Forward)	CCAGGGTATGGACCTGTCTG
FSCN1 (Reverse)	CGCCACTCGATGTCAAAGTA
**ChIP qPCR primer name**	**Sequence (5′–3′)**
LOC146880 promoter-F	TTTTCCACCCTGGCCACAAGGC
LOC146880 promoter-R	TTCCTCTCTCAGGGTCTCGGTTT
**Silencing RNA name**	**Sequence (5′–3′)**
LOC146880 si#1	GACCAAGACUACCCACAAUCUTT
LOC146880 si#2	GCGACUCAGAGAACGUCUAAGTT
LOC146880 si#3	GGCAGAUUUAGUUAAGCUCUCTT
FSCN1 si#1	CCCUUGCCUUUCAAACUGGAAdTdT
miR-328-5p mimics	GGGGGGGCAGGAGGGGCUCAGGG
miR-328-5p inhibitor	CCCUGAGCCCCUCCUGCCCCCCC
**Short harpin RNA name**	**Sequence (5′-3′)**
LOC146880 shRNA	CCGGGCGACTCAGAGAACGTCTAAGTTCTCGAGAACTTAGACGTTCTCTGAGTCGCTTTTTG
